# Extended Lifetime of Molecules Adsorbed onto Excipients Drives Nucleation in Heterogeneous Crystallization

**DOI:** 10.1021/acs.cgd.0c01532

**Published:** 2021-03-11

**Authors:** Pierre-Andre Cazade, Vivek Verma, Benjamin K. Hodnett, Damien Thompson

**Affiliations:** † Department of Physics and the Synthesis and Solid State Pharmaceutical Centre, Bernal Institute, University of Limerick, Limerick V94 T9PX, Ireland; ‡ Department of Chemical Sciences and the Synthesis and Solid State Pharmaceutical Centre, Bernal Institute, University of Limerick, Limerick V94 T9PX, Ireland

## Abstract

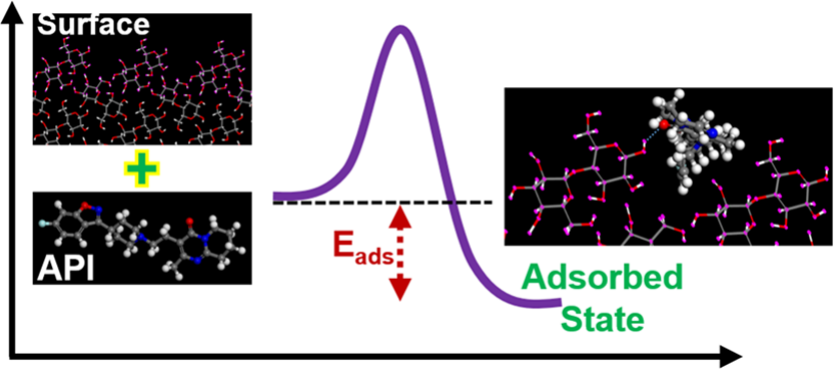

Monte Carlo (MC) and molecular dynamics (MD) computer simulations were used to investigate the role of adsorption during seeded and heterogeneous crystallization. The simulations characterized the range of adsorption energies and configurations encountered during adsorption of individual molecules of active pharmaceutical ingredients (APIs), with varying hydrogen-bonding tendencies, onto seed and heterosurfaces. Specifically, the adsorption of acetaminophen (AAP), carbamazepine (CBMZ), fenofibrate (FF), phenylbutazone (PBZ), clozapine (CPB), and risperidone (RIS) was simulated on selected crystallographic facets of their own crystals as examples of seeded crystallizations and on lactose or microcrystalline cellulose (MCC) substrates as heterosurfaces. The MC screening provided adsorption enthalpies in the range of −59 to −155 kJ mol^–1^ for these APIs on lactose, generally increasing as the molar mass of the API increased. The corresponding values predicted for adsorption of each API onto its own crystal were in the range of −92 to −201 kJ mol^–1^. More detailed MD simulations performed in methanol showed adsorption free energies for RIS on MCC in the range of −37 to −50 kJ mol^–1^ with strong molecule–surface complexation lifetime of tens of nanoseconds on the (010) face of MCC. This extended lifetime is a key feature in understanding the mechanism of heterogeneous crystallization. A well-formed nucleus is generated on the surface starting with a single adsorbed molecule. Individual or small clusters add to the adsorbed species. This addition is facilitated by the extended lifetime of the adsorbed molecule, which is several orders of magnitude greater than the time required for additional molecules to assemble and grow into a stable nucleus attached to the heterosurface.

## Abstract 2

In heterogeneous crystallization, a well-formed nucleus is generated on the surface starting with a single adsorbed molecule. The addition of molecules is facilitated by the extended lifetime of the adsorbed molecule, allowing additional molecules to assemble and grow into a stable nucleus attached to the heterosurface.

## Introduction

Heterogeneous nucleation is the process in which crystallization is initiated by the interaction of gas phase or solute molecules with foreign solids (not seed). Heterogeneous nucleation is ubiquitous in the natural environment and is, for example, associated with ice nucleation in the atmosphere. Mineral dust particles are thought to be the most prevalent ice nucleation particles (INPs). Heterogeneous nucleation of ice seems to be particularly important in circumstances where supersaturation is low. Two mechanisms are commonly cited, immersion and contact freezing. In immersion, INPs induce freezing inside a water droplet, whereas contact freezing occurs on the surface of the droplet.^1^

The subject of heterogeneous nucleation for organic molecules was put on a modern scientific footing by Ward and co-workers.^2−4^ They used freshly cleaved single crystals such as β-succinic acid or l-valine as substrates for the nucleation of benzoic acid delivered to the substrate by sublimation. This work led to the concept of ledge-directed epitaxy (LDE) where the nucleation site was postulated to be a pair of intersecting cleavage planes on the substrate, which generated a good lattice match with the nucleating benzoic acid.^2^

Subsequently, the subject has expanded to include compounds and conditions that are more interesting for pharmaceutical applications.^5^ The assembly of the molecules on heterosurfaces may occur through interactions such as functional group matching,^6^ lattice matching,^6^ or through nonspecific adsorption of the molecules on the heterosurface.^7^ Self-assembled monolayers,^8−10^ silanized glass substrates,^11−13^ pharmaceutical excipients,^6,14−16^ biocompatible polymers,^17,18^ synthetic polymers,^19−24^ and porous substrates^25^ have been used as heterosurfaces to induce nucleation. Chemically modified surfaces have also been used for protein crystallization.^26^ There are several examples in the literature of stable phases nucleating on the surface of metastable phases during solution-mediated polymorphic transformations.^27−30^

Of specific interest for the current study is the report by Diao et al.^31^ that examined the nucleation density of aspirin on a range of polymers. Two polymers, in particular, poly(4-acryloylmorpholine) and poly(2-carboxyethyl acrylate), showed a dramatic increase in nucleation density. This is because the hydrogen bond donor (HBD) sites of the aspirin could interact most effectively with the hydrogen bond acceptor (HBA) sites on these polymers. In addition, the (011) facet of aspirin, which features free carboxylic acid terminations, always attached to the surface of these polymers in a clearly orientated manner.

Diao et al.^32^ also engineered pores in polymers of differing shapes (round, square, and hexagonal) generating different angles between the pore walls. Polymers with hexagonal pores accelerated the crystallization of aspirin more than square or round pores because of a favorable matching between the pore wall angles and the faces of the nucleating compound. While porosity greatly accelerated the nucleation rate in polymers that already possessed the requisite complementary HBA properties, porosity was not effective for polymers that did not possess HBA capacity.

Heterogeneous nucleation of acetaminophen (AAP) has received a lot of attention. Substrates studied include poly(methylmethacrylate) (PMMA), poly-*n*-butylmethacrylate (PBMA), δ-mannitol, α-lactose monohydrate, graphite, and l-histidine.^6,33^ Aspects investigated include the hydrogen-bonding complementarity between AAP and the substrate and the role of lattice matching. Both were considered important but the role of hydrogen bonding was considered more important. Graphite was a particularly interesting heterosurface as it was among the poorest in accelerating heterogeneous nucleation.^6,33^

In a study by Arribas Bueno et al.,^15^ fenofibrate (FF) was examined because it does not contain any groups capable of acting as HBD sites. Its crystallization from methanol was carried out in the presence of α/β-lactose (α/β-Lac), δ-mannitol (d-Man), microcrystalline cellulose (MCC), carboxymethyl cellulose (CMC), silica (SiO_2_), and poly(caprolactone) (PCL). Each of these excipients except PCL features multiple HBDs, and all but PCL were effective in strongly reducing the FF induction time. In this work, the authors suggested that adsorbed compounds attached to a heterosurface exhibit a much longer lifetime than the corresponding compounds attached to each other in the solution phase. The significance of the longer lifetime of adsorbed compounds is that it allows sufficient time for multiple molecules of the same compound to interact with and become attached to the adsorbed molecule or a developing nucleus.

A further study by Verma et al.^34^ examined the acceleration of the crystallization of seven APIs in the presence of a single excipient, microcrystalline cellulose (MCC), which features multiple HBD groups. Five of the compounds, caffeine (CAF), phenylbutazone (PBZ), risperidone (RIS), clozapine (CPB), and fenofibrate (FF), exhibit HBA properties only. The remaining two, acetaminophen (AAP) and carbamazepine (CBMZ), exhibit both HBD and HBA properties. Crystallization of the five APIs with HBA functionality only was strongly accelerated when MCC was present during the crystallization (up to 16 times faster), whereas the two compounds with HBA and HBD properties exhibited a much more modest acceleration (less than ×2) for the same conditions. Scanning electron microscopy (SEM) analysis of the API–MCC composite powders confirmed that all API particles attached to the MCC carrier particles. This study confirmed the benefits of hydrogen-bonding complementary (HBD in the excipient and HBA in the APIs in this study) but a key finding was the degree of attachment of AAP and CBMZ to the excipient. This feature was identified as an important argument for the extended lifetime of adsorbed compounds, created from the adsorption of single molecules or small clusters, allowing sufficient time for other API molecules to interact and coalesce into stable nuclei that remained attached to the heterosurface and eventually grew into fully stable crystals.

The field of adsorption is also of interest in developing a theory of heterogeneous nucleation. Traditionally, this field has concentrated on the adsorption of small molecules such as CO, NO, and H_2_O onto metals (*e.g*., Pt, Ni) and metal oxides (*e.g*., SiO_2_, Al_2_O_3_, MgO, and many others).^35−37^ Of particular interest for the present study is the recent work that examines the adsorption of small organic molecules onto metal oxide surfaces. These reports typically combine infrared spectroscopy and temperature-programmed desorption studies with molecular simulations to determine adsorption configurations, enthalpies of adsorption, and, in some cases, adsorption lifetimes. In one representative example, the adsorption of α-pinene onto fused silica was studied,^38^ with the calculated enthalpy of adsorption of −39 kJ mol^–1^ and the free energy of adsorption of −22 kJ mol^–1^. Molecular dynamics (MD) studies indicated that limonene adsorbed onto silica in several possible configurations, most of which exhibited one π-hydrogen bond to the silica surface.^39^ For limonene on SiO_2_, the enthalpy of adsorption was −55 kJ mol^–1^ and the free energy of adsorption was −30 kJ mol^–1^. These values are entirely consistent with the desorption energies in the range of −31 to −47 kJ mol^–1^ reported for a range of substituted benzene compounds (toluene, iodobenzene, chlorobenzene, *etc*.) on silica.^40^ A more general and comprehensive compilation of enthalpies of adsorption, principally for hydrocarbons and alcohols onto a range of metal oxides, showed that values were typically between −50 and −150 kJ mol^–1^, generally increasing as the molecular size increased.^41^ Recent MD modeling studies have quantified the roles of solvation and heterostructure in determining the strength of the binding free energies of a variety of molecule–substrate complexes.^42,43^

Here, we systematically probe the nature of the interactions between a heterosurface and a range of API molecules, some of which feature HBD and HBA properties and some HBA properties only. The approach was to treat the first nucleation interactions between an API and a heterosurface as an adsorption phenomenon. The adsorption properties of the full range of APIs tested experimentally in ref (34) were screened using the Monte Carlo (MC) method to generate adsorption complexes and estimate binding enthalpies for each API on a lactose substrate selected for its abundance of HBD sites on each Miller surface. Lactose was an effective surface in promoting heteronucleation in our previous work.^14−16^ The MC method returned multiple low-energy adsorption configurations (generally >10) for each API adsorbed on lactose. The rapid MC screen was complemented by detailed molecular dynamics (MD) simulations of a selected API–excipient combination from ref (34) namely, RIS adsorbed onto methanol-solvated microcrystalline cellulose (MCC). Both of the excipients examined here are commonly used in pharmaceutical formulations and are sometimes used as a blend.^44^

## Methods

The Monte Carlo method^45,46^ was applied as a screening tool to generate adsorption configurations and enthalpies for a range of APIs on a model lactose surface. Six APIs were selected for this study. AAP and CBMZ possess both HBD and HBA functional groups, while FF, PBZ, CPB, and RIS only possess HBA functional groups. Since methanol was the only solvent used in ref (34) its adsorption onto lactose was also evaluated using the MC method. Including the solvent provides a more accurate model of the binding process and the competition that can exist with solvation. A recent study of the crystallization of salicylic acid in a range of solvents suggests that nucleation becomes more difficult as the binding between the solvent and the solute becomes stronger.^47^

MC simulations were carried out using the adsorption locator module of Materials Studio Version 7.0. Crystal structures were generated from the CCDC database with the aid of the Mercury Software 2020.1. In a first step, the required adsorbate (API or solvent) was drawn and its geometry optimized using the Forcite Compass II force field. Electrostatic and van der Waals (vdW) interactions were truncated at their default 1.25 nm cutoffs to enable high-throughput screening. The required adsorbent structures were selected from the CCDC database. Miller planes were selected based on the most prominent BFDH areas identified by the Bravais, Friedel, Donnay, and Harker crystal morphology method in the Mercury software. The adsorbent model was created from a 64-unit supercell of the crystal structure with the unit cells generally arranged in an 8 × 4 × 2 array in the *abc* directions with the selected Miller plane exposed to a vacuum slab of 1.5 nm. To facilitate broad and rapid screening, explicit bulk methanol solvent molecules were not included in the model. The calculation of adsorption configurations with the lowest adsorption energies (*E*_ads_) was carried out using the adsorption locator module with the Compass II force field for both the geometry optimization and the adsorption energy calculations. This force field has been successfully used in the past for small drug molecules.^34,46^

Adsorption locator generates adsorption configurations *via* MC searches of the configurational space of the adsorbate–adsorbent complexes with simulated annealing used to slowly lower the temperature and identify the most stable binding sites. A fraction of the exposed crystal structure was selected as the target to which adsorption was confined. The maximum allowed adsorption distance was set at 0.5 nm. The simulated annealing calculation was performed over 4 heating cycles with 5000 steps per cycle. For each simulation, this number of loading steps was confirmed as sufficient for the energy optimization.

Each calculation returned a number of adsorption configurations and an estimate of the *E*_ads_ for each configuration. The 10 configurations with the lowest *E*_ads_ values were selected for each adsorbate–adsorbent complex and examined in detail to identify if there was a hydrogen-bonding component of the total adsorption energy. Note that neither COMPASS nor CHARMM (see below) force fields retain a specific interaction potential dedicated to hydrogen bonding, distinct from other sources of electrostatic and van der Waals’s interactions.

To benchmark the reliability of the high-throughput MC screens, larger-scale molecular dynamics (MD) simulations were performed on a representative adsorbate–adsorbent complex in full explicit solvent. The simulation cell contained a single RIS molecule bound to an MCC particle surrounded by 9891 methanol molecules, in a periodic box that reproduced the bulk density of methanol at an ambient temperature and pressure. We used the Gromacs 2018.4 code to calculate dynamics. The CHARMM^48^ force field for carbohydrates was used to model MCC and its complementary generalized force field for small organic molecules GGenFF^49^ was used to model methanol and RIS, with RIS parameterized using the ParamChem server. Two standard force fields are commonly used for the modeling of small organic molecules including APIs: CGenFF that was developed to be compatible with the CHARMM force field and GAFF, which was developed to be compatible with the AMBER force field. Both CHARMM and AMBER were initially developed for the modeling of proteins in solution but have since been extended to the modeling of DNA/RNA, lipids, and carbohydrates. Having access to reliable models for sugars has been crucial in selecting a force field, as most excipients are sugar-based.

All bonds to hydrogen were constrained using the LINCS^50^ algorithm, which allowed an integration time step of 2 fs. The MD trajectory was calculated using the leapfrog integrator,^51^ and coordinates were saved every 2 ps. Long-range electrostatics were treated by the particle mesh Ewald (PME) method, which calculates all electrostatic interactions in the periodic simulation cell.^52^ The standard truncation in the CHARMM of 1.2 nm was used for vdW interactions.

All systems were minimized for 10 000 steps and heated progressively from 0 to 300 K for a total of 0.5 ns at a constant volume–temperature (NVT), followed by 0.5 ns of a constant pressure (NPT) equilibration. The reference temperature was set at 300 K with a time constant of 1 ps, and the reference pressure was set at 1 bar with a time constant of 5 ps, using the Berendsen^53^ thermostat and barostat, respectively. Following thermalization and equilibration, the subsequent production phase of dynamics (in which the total energy, temperature, and pressure have plateaued and properties can be estimated) was carried out in the NPT ensemble. The reference pressure was set at 1 bar with a time constant of 5 ps using the Parrinello–Rahman barostat,^54,55^ and all molecules coupled separately in groups to an external heat bath were set at 300 K with a coupling time constant of 1 ps using the v-rescale^56^ method. All analyses were performed with Gromacs tools, and trajectories were visualized using visual molecular dynamics (VMD),^57^ a standard software for preparation, visualization, and analysis of MD simulations.

We simulated the adsorption of RIS on different faces of a block of methanol-solvated MCC. In all simulations, the RIS molecule is initially positioned and oriented in a binding orientation conducive to strong hydrogen bonding with each heterosurface.

## Results

Table 1 shows the chemical structure of the six API molecules examined in this study, together with a summary of the numbers of hydrogen bond donor (HBD) and hydrogen bond acceptor (HBA) groups in each molecule. HBA and HBD sites in the APIs were identified and counted using the hydrogen bond propensity module of the Mercury 2020.1 code. AAP, CPB, and CBMZ each possess HBD and HBA groups, whereas RIS, PBZ, and FF possess only HBA groups. As reported by Verma et al.,^16^ the methanol solvent can competitively bind at the API–excipient interface, *e.g*., blocking the only HBD site of CPB to leave CPB with only HBAs to hydrogen-bond with lactose.

**Table 1:**
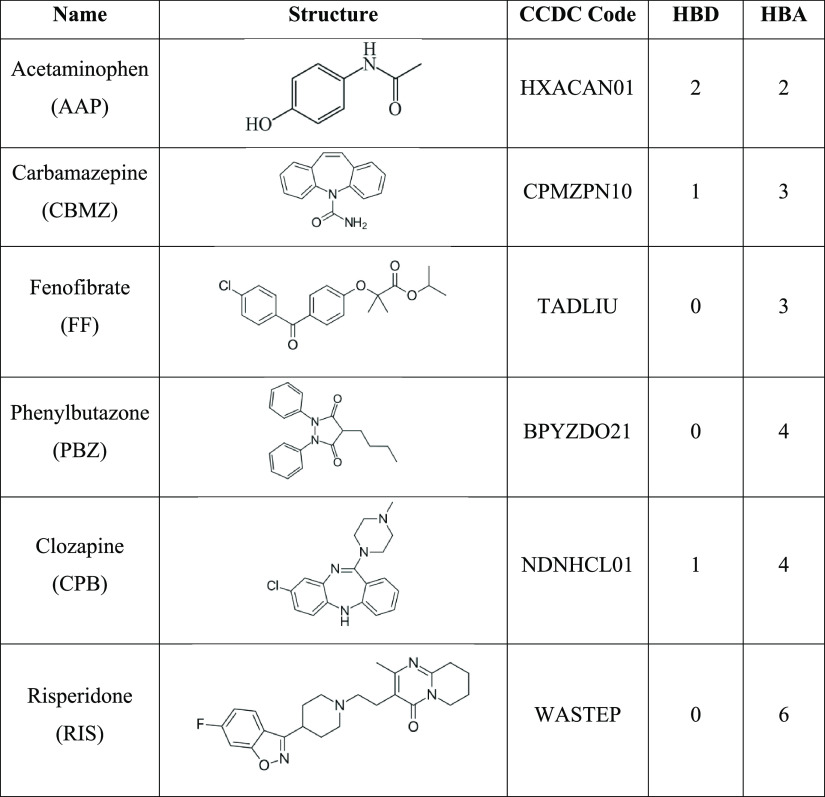
Chemical Structures of the Six APIs Used in This Study with the Number of Hydrogen Bond Donor (HBD) and Hydrogen Bond Acceptor (HBA) Groups in Each Structure Shown

Table 2 presents the calculated *E*_ads_ values for methanol and AAP adsorbed from the gas phase onto the indicated cleavage planes of lactose. Lactose was selected as the adsorbate because of its extensive use as an excipient and the large concentration of HBD groups on each of its cleavage planes.

**Table 2:** Adsorption Energies for Methanol and AAP on Various Lactose Planes (CCDC Code BLACTO)^a^

lactose (*hkl*)	BFDH relative area	*E*_ads_ methanol/kJ mol^–1^	*E*_ads_ AAP/kJ mol^–1^
(100)	0.296	–71 to −46	–125 to −105
(001)	0.130	–84 to −54	–163 to −142
(020)	0.108	–79 to −50	–159 to −130
(111)	<0.001	–79 to −50	–138 to −113
(110)	0.178	–67 to −46	–146 to −117

a The range of *E*_ads_ values spans the 10 lowest energy configurations.

All adsorption sites returned a significant *E*_ads_ for methanol adsorbed onto all cleavage planes of lactose examined (in the range of −46 to −84 kJ mol^–1^) and distinctly larger values for AAP adsorbed onto lactose (in the range of −105 to −163 kJ mol^–1^). The *E*_ads_ ranges returned are not strongly affected by the lactose cleavage plane for methanol and AAP. The range of values we report in Table 2 (and in Table 3) represents the 10 lowest *E*_ads_ for 10 separate adsorption configurations for a particular API adsorbed onto a specific crystallographic plane of lactose, indicating that these ranges reflect the Boltzmann distribution (also called Gibbs distribution) of adsorption configurations and enthalpies that are accessible to each API on the excipient surface.

**Table 3:** *E*_ads_ for API onto Lactose (100), on a Selected Miller Plane of Its Own Structure and for Methanol on the API^a^

adsorbate	*E*_ads_ API on lactose (100)/kJ mol^–1^	adsorbent (*hkl*)	*E*_ads_ API on the indicated API (*hkl*)/kJ mol^–1^	*E*_ads_ methanol on the indicated API (*hkl*)/kJ mol^–1^
AAP	–125 to −105	AAP (110)	–105 to −79	–63 to −33
CBMZ	–79 to −59	CBMZ (01̅1)	–113 to −100	–54 to −38
FF	–109 to −96	FF (001)	–117 to −100	–33 to −22
PBZ	–100 to −96	PBZ (001)	–142 to −130	–46 to −25
CPB	–105 to −88	CLZ (110)	–113 to −96	–50 to −38
RIS	–155 to −142	RIS (101̅)	–201 to −188	–54 to −42

a The range of *E*_ads_ values spans the 10 lowest energy configurations.

The absolute values of these adsorption energies are sizable when compared to the typical values of approximately −200 kJ mol^–1^ for chemisorption of small molecules onto metal surfaces.^58^ They are also consistent with a recent experimental and MD study of limonene on SiO_2_ that reports *E*_ads_ values in the range of −50 to −60 kJ mol^–1^ and an extensive review of small organic molecules (hydrocarbons, alcohols, ketones, and carboxylic acids) adsorbed onto a range of metal oxides with adsorption energies of −20 to −150 kJ mol^–1^.^36,37,39,41^

**Figure 1:**
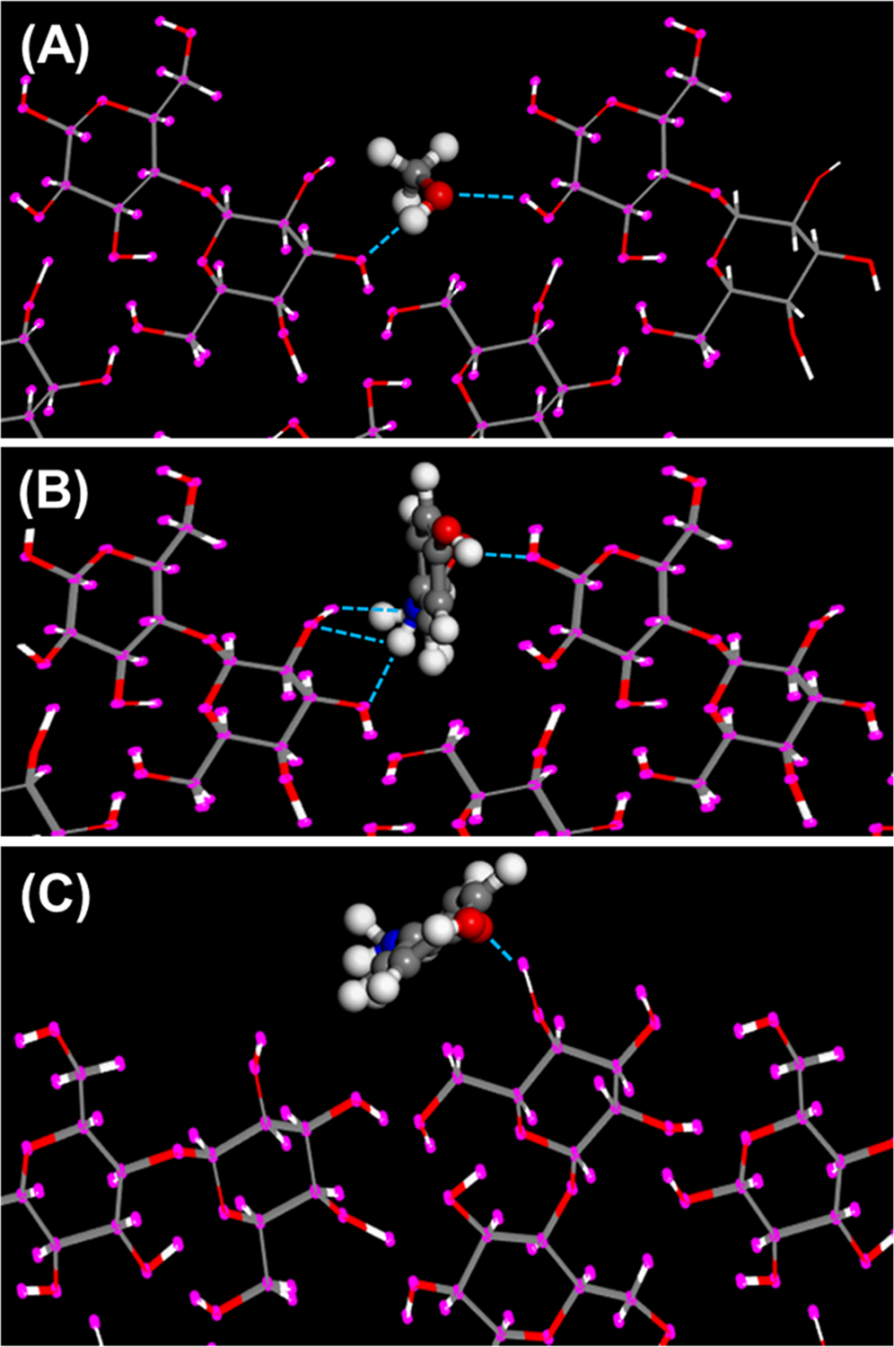
Mode of adsorption of (A) methanol onto lactose (100), (B) AAP onto lactose (100), and (C) AAP onto lactose (110). The blue dotted lines represent hydrogen bond contacts with a length less than 0.25 nm. For clarity in this figure and in Figures 2–6, the adsorbate (API or solvent) is presented as a CPK ball-and-stick model and the adsorbent is shown in thin-stick representation.

Figure 1 presents some representative modes of adsorption of methanol and AAP onto the indicated cleavage planes of lactose. All modes of adsorption for methanol and AAP on lactose featured single or multiple strong hydrogen bonds with lengths ≤0.25 nm, leading to the conclusion that all lactose cleavage planes provide hydrogen-bonding interaction sites for methanol and AAP.

Table 3 presents the range of *E*_ads_ values returned for the six APIs on the (100) plane of lactose (column 2), which is its largest BFDH fractional plane. Also given are the adsorption energies for the six APIs adsorbed onto the largest BFDH fractional plane of their own crystal structures (columns 3 and 4) and for the adsorption of methanol onto the same plane of each of the six APIs (column 5).

The results in column 2 of Table 3 show that all of the APIs tested in this way demonstrate strong adsorption on lactose (100) with adsorption energies entirely consistent with literature reports.^36,37,39,41^ In five of the six cases studied, adsorption energies are slightly weaker on lactose (100) than the corresponding energies for adsorption of these APIs onto selected faces of their own crystal structures (column 4, Table 3). A further feature is that the *E*_ads_ values for methanol on the six selected faces of the API crystal structures were slightly smaller than the *E*_ads_ values for methanol adsorbed onto the various planes of lactose (Table 2).

**Figure 2:**
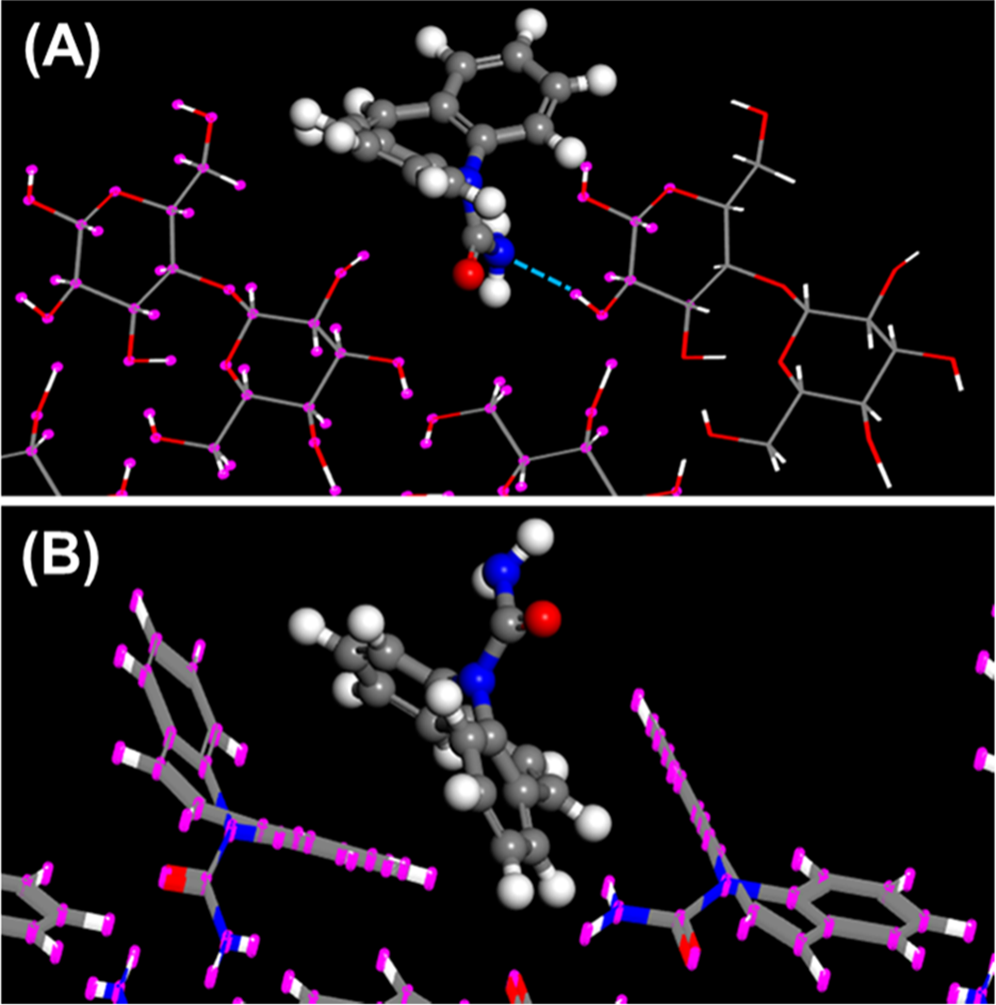
Mode of adsorption of (A) CBMZ onto lactose (100) and (B) CBMZ onto CBMZ (0̑̑̑1̅1).

**Figure 3:**
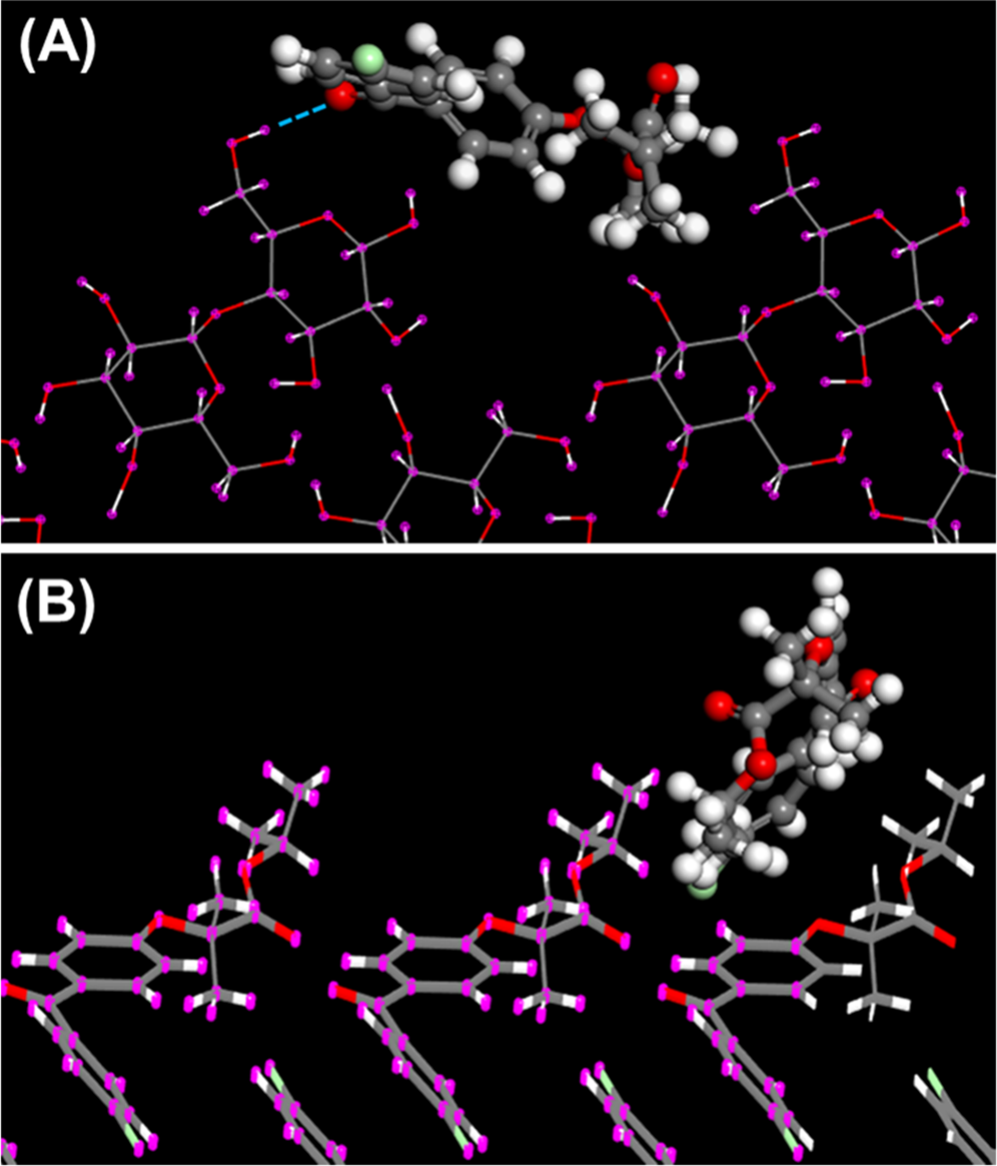
Mode of adsorption of (A) fenofibrate onto lactose (100) and (B) fenofibrate onto fenofibrate (001).

**Figure 4:**
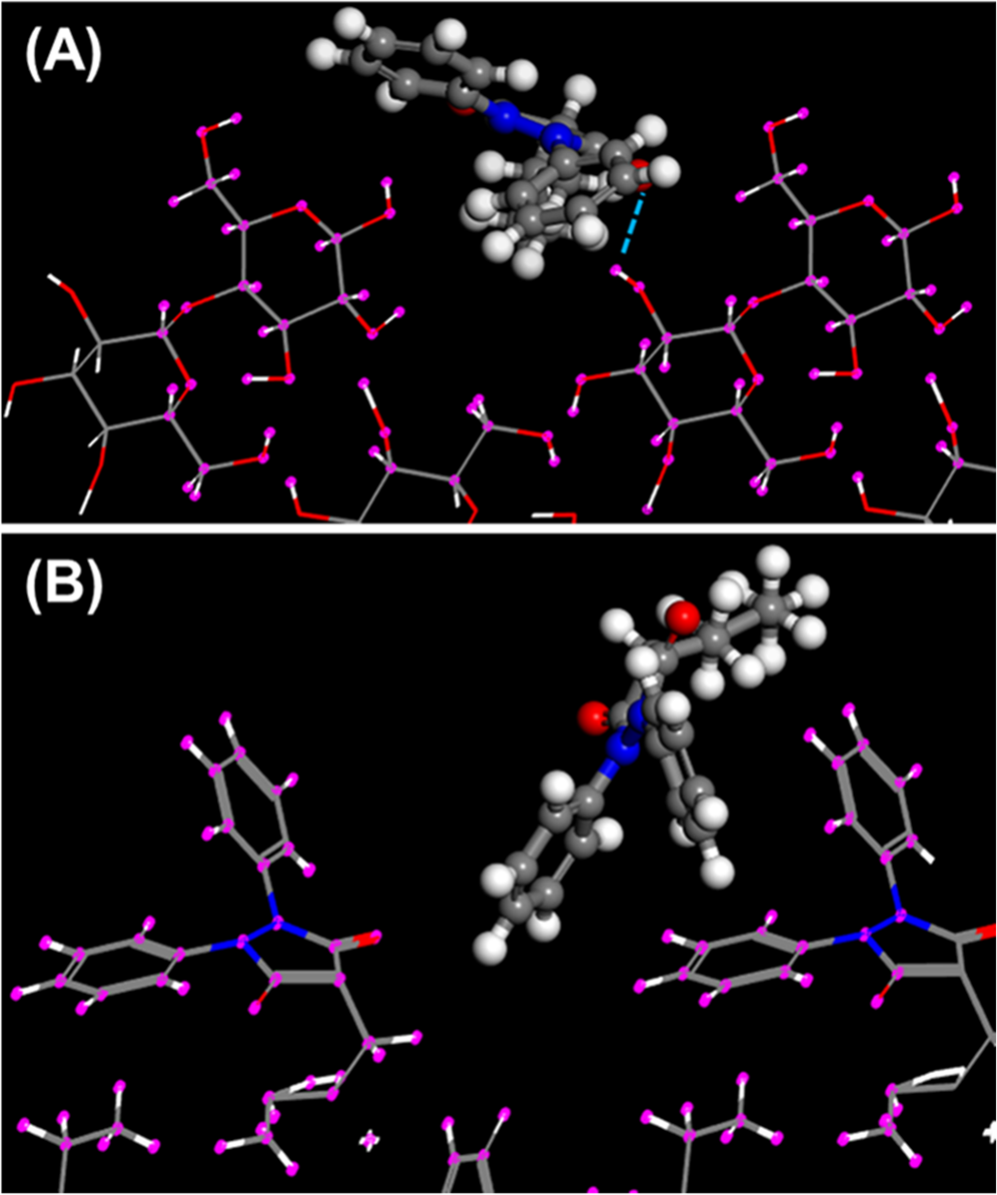
Mode of adsorption of (A) phenylbutazone onto lactose (100) and (B) phenylbutazone onto phenylbutazone (100).

**Figure 5:**
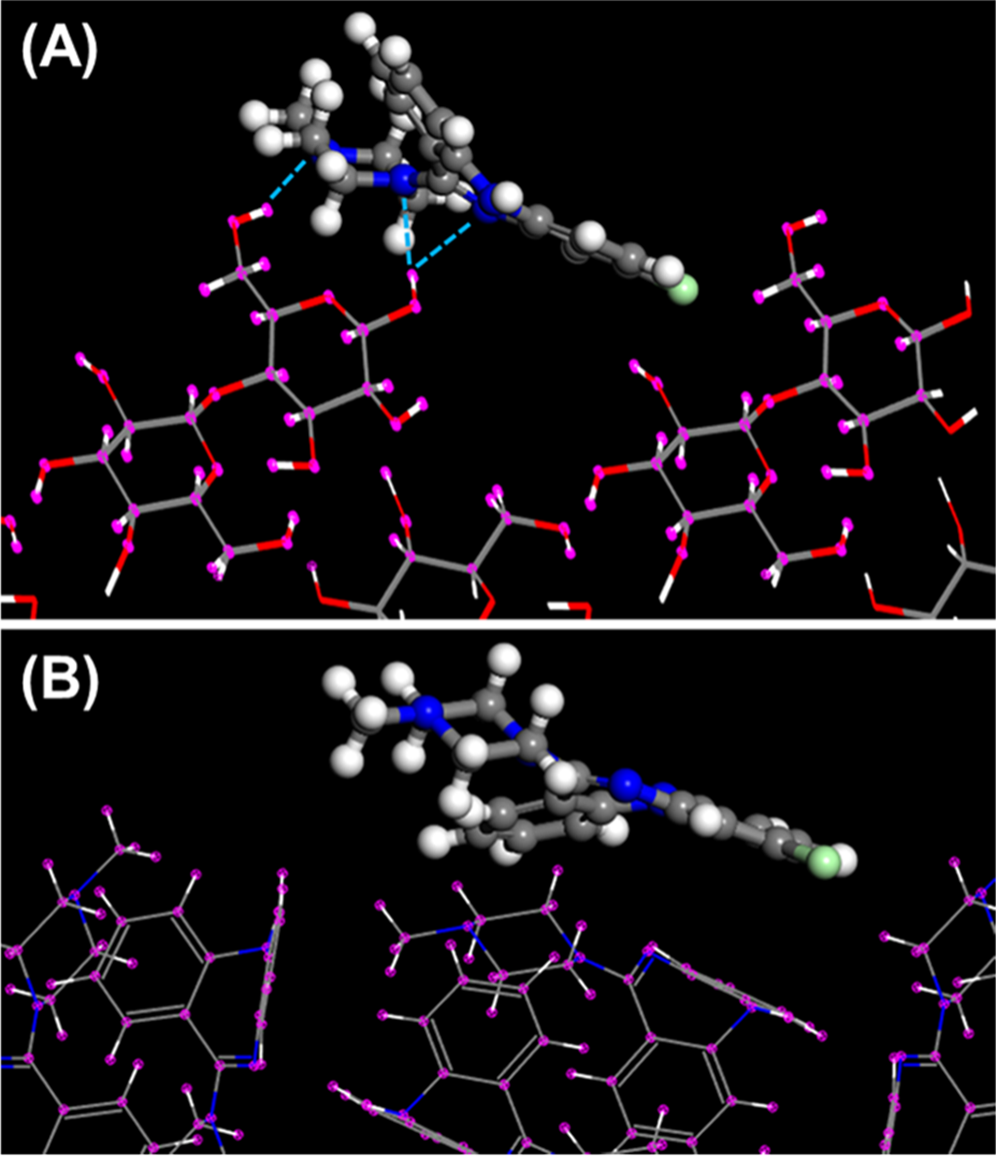
Mode of adsorption of (A) clozapine onto lactose (100) and (B) clozapine onto clozapine (110).

**Figure 6:**
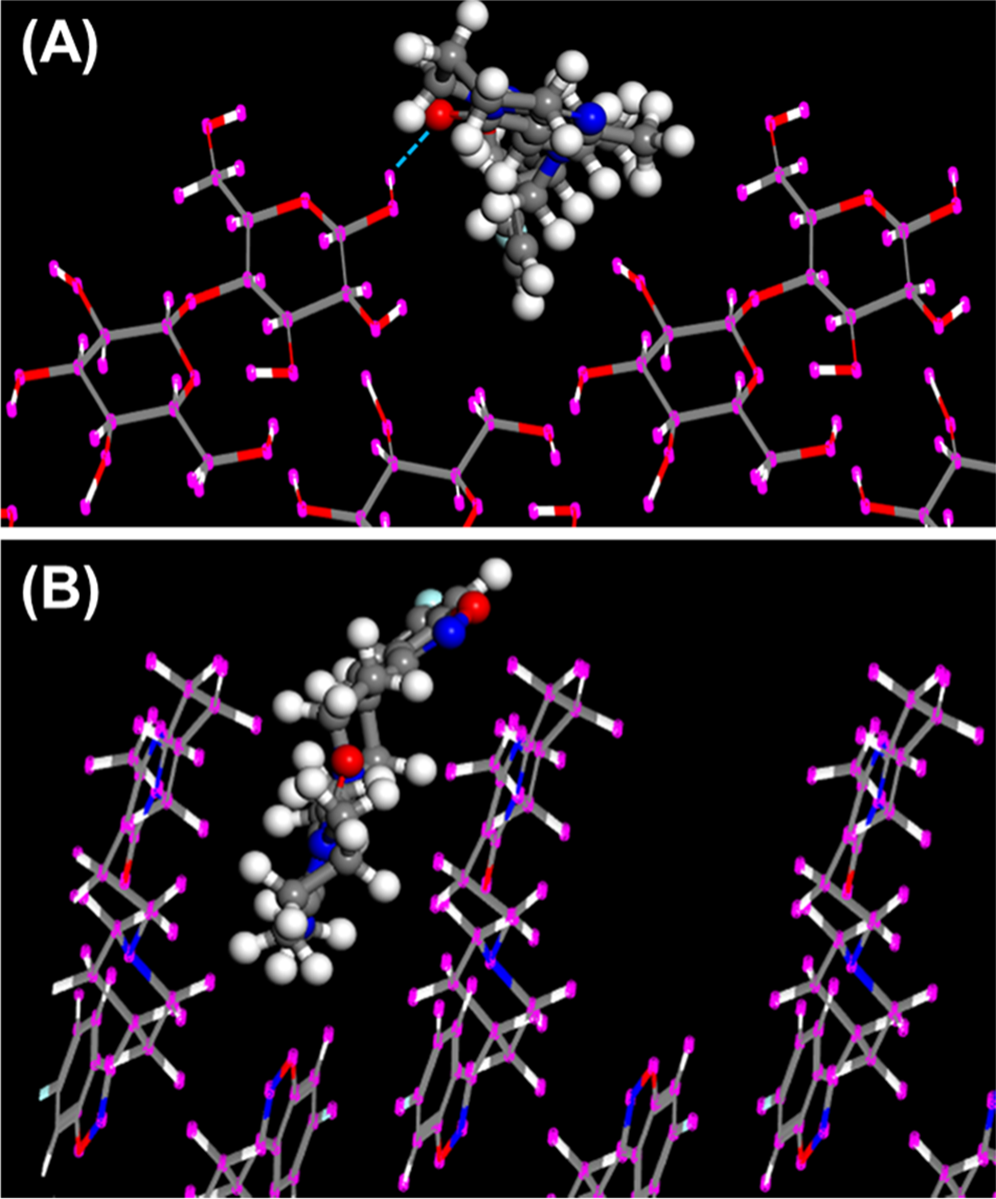
Mode of adsorption of (A) risperidone onto lactose (100) and (B) risperidone onto risperidone (101̅).

Further detailed investigation demonstrates that hydrogen bonding occurs between the selected APIs and lactose including those with only HBA groups (Table 2). Standard criteria are used to determine the number of H-bonds between two species. The algorithm looks for donor and acceptor polar atoms (N, O, S, F, and some C). To act as a donor, the atom must carry a hydrogen atom. Then, a H-bond exists if the D–A distance is <0.3 nm and the D–H–A angle is <20°. Our simulations confirm that, as expected, there is no hydrogen bonding for any API that only possesses HBA groups when adsorbed onto its own crystal structure. This point is further illustrated in Figures 2–6. The top graphic in each figure presents the lowest energy site for adsorption of the selected API onto lactose (100). In each case, at least one H-bond can be identified between the API and the lactose (100) surface. This finding was confirmed for each of the 10 lowest energy adsorption sites for all of the APIs listed in Table 3 for adsorption onto lactose. On the other hand, the analysis confirmed that the APIs that do not possess HBD groups (FF, PBZ, and RIS) did not exhibit any hydrogen bonding when adsorbed onto their own crystal structure, as expected. Surprisingly, in the cases of CBMZ and CPB, each of which possesses one HBD group, hydrogen bonding was not observed when each molecule adsorbed onto the selected plane of its own crystal structure. This indicates that hydrogen bonding is not essential for crystal growth. It is known that a new secondary nucleus can develop from the adsorbed state, especially through the crystal breeding mechanism.^59^ Future works could systematically scan API molecule adsorption on all energetically stable API crystal faces to further map the potential energy surface for this self-interaction between the molecule and seed.

**Figure 7:**
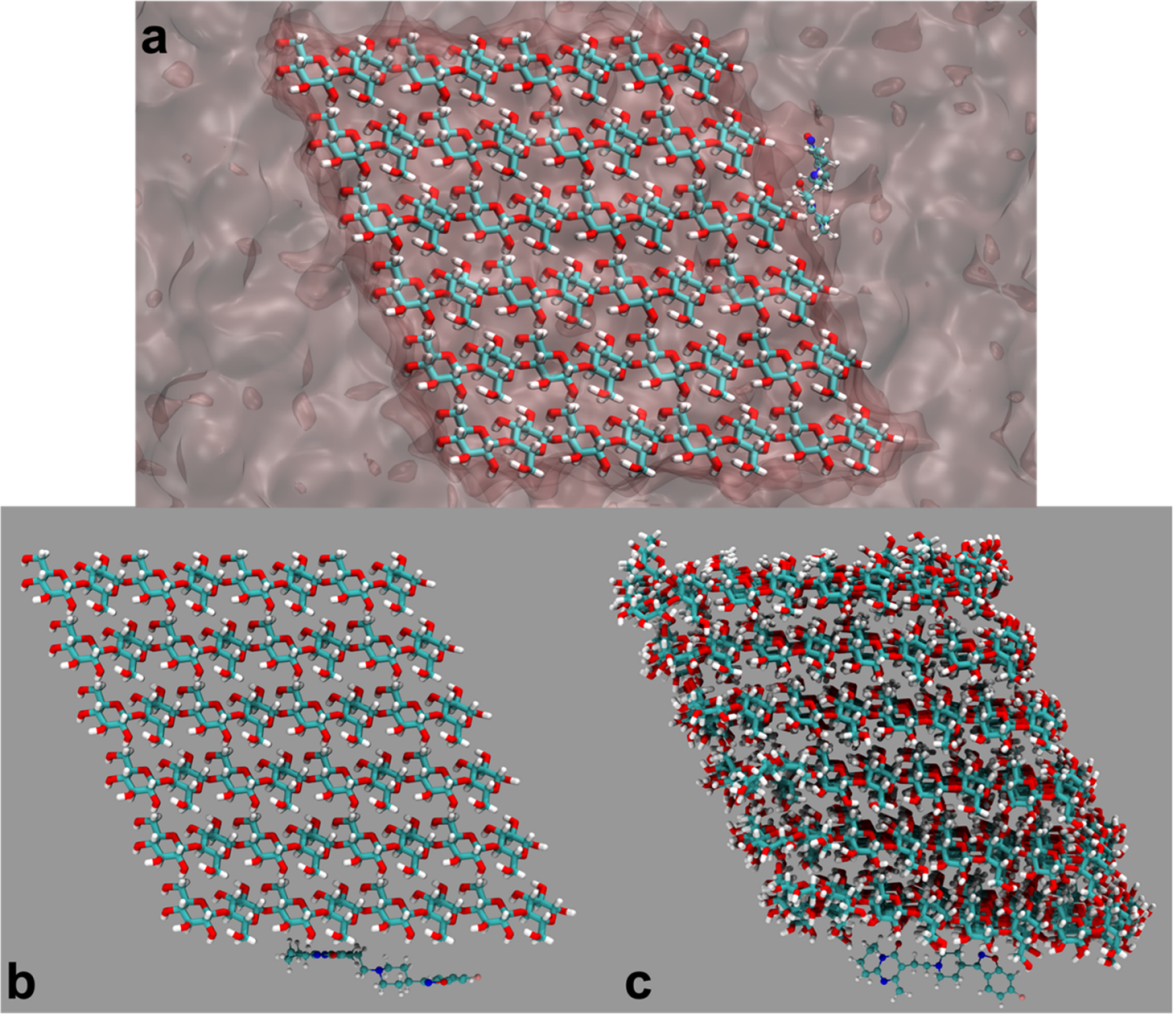
(a) Starting configuration of RIS on the (100) face of MCC for MD simulations in bulk methanol. MCC atoms are shown as thick sticks, RIS as balls and sticks, and the explicit methanol bulk solvent molecules are drawn as a semitransparent surface. Red balls/sticks represent oxygen atoms, nitrogen atoms are navy blue, carbon atoms are green, and hydrogen atoms are white. (b) Starting and (c) typical bound structure during strong binding dynamics of RIS on the (010) face of microcrystalline cellulose. Methanol solvent is not depicted for the sake of clarity.

**Figure 8:**
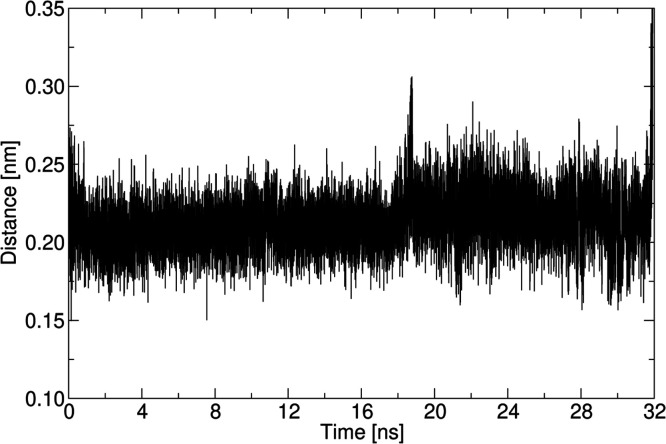
Contact distance between RIS and the (010) face of MCC in methanol during the 32 ns of strong complexation.

In addition to lactose, microcrystalline cellulose (MCC) is another promising candidate for adsorption of APIs as it presents potential H-bond sites on many of its faces. We performed three series of MD simulations with RIS placed initially within the binding distance of different faces of MCC in bulk methanol (Figure 7). In two of the simulations, for interaction with the (100) and (001) faces of the crystal, RIS did not stick to the surface. However, it bound strongly to the (010) face for tens of nanoseconds of unconstrained well-equilibrated room-temperature dynamics. Figure 8 shows the shortest distance between atoms of RIS and MCC during ∼30 ns of strong binding, confirming that the API remains within a close hydrogen bond contact distance with MCC throughout. The movie in the Supporting Information shows that RIS maintains a looser association with the surface for another ∼65 ns before completely desorbing.

**Figure 9:**
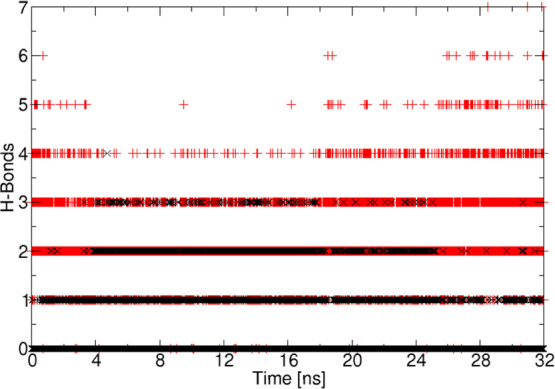
H-bond population dynamics during 32 ns of strong binding between RIS and MCC (black) and between RIS and methanol (red).

To further characterize the interaction between RIS and the (010) face of MCC, we monitored the number of H-bonds over time. We compare the RIS–MCC binding with the H-bonding between RIS and methanol as the balance between adsorption and desorption is determined by the competition between the API/substrate and the API/solvent interactions (Figure 9). On average, RIS forms more H-bonds with methanol than with MCC. However, as the simulation progresses, the number of H-bonds with methanol decreases to stabilize between two and three, while the number of H-bonds with cellulose increases to stabilize between one and two. Finally, the number of H-bonds between RIS and methanol increases again, while the number of H-bonds with cellulose decreases leading to the unbinding of RIS. This finding emphasizes the importance of the choice of solvent as well as maximizing API–excipient H-bonding in designing heterogeneous crystallizations. Taken together, the data in Figures 8 and 9 confirm that the RIS remains strongly bound *via* H-bonds to the excipient surface for the full 32 ns of strong binding dynamics. The MD-calculated H-bound adsorption lifetime is consistent with a recent study of water adsorption onto mesoporous silica. In that work, lifetimes close to 20 ns were reported for adsorbed water.^37,39^

**Figure 10:**
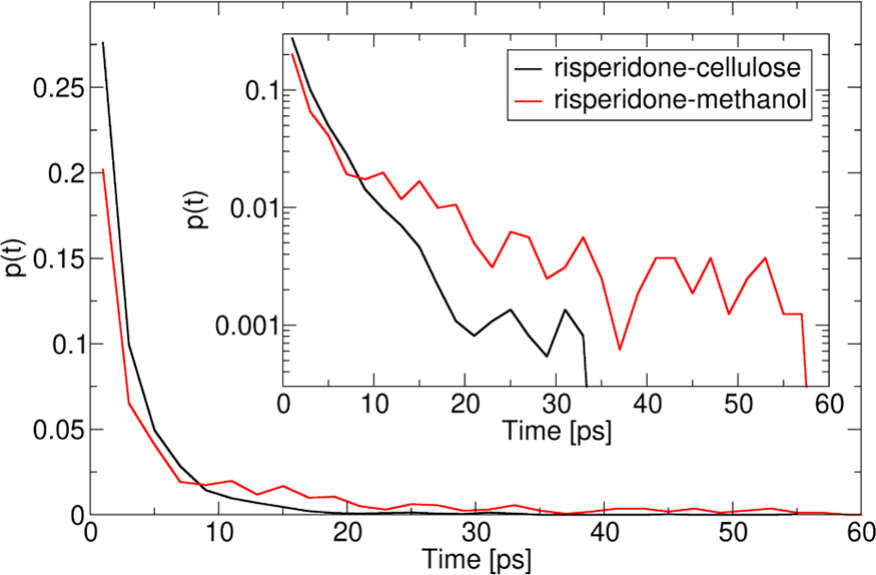
Individual pairwise H-bond lifetimes for RIS–MCC (black) and RIS–methanol (red).

Figure 10 shows the average lifetimes of the individual specific pairwise H-bonds that RIS forms with cellulose and with methanol. The lifetimes are similar, with H-bonds to cellulose slightly longer-lived than H-bonds to methanol. The lifetime of the individual H-bonds is sub-10 ps, which is typical of H-bonds in general, *e.g*., H-bonds in liquid water exchange every 10 ps or so.^60,61^ This emphasizes that the overall strong interaction is summed over a large population of short-lived interactions. Biology uses similar multisite or multivalent attachment of large molecules to receptor surfaces through multiple, individually weak, reversible interactions. This provides overall binding energies as strong as chemisorption, yet the complexes can be dissociated by simply changing the pH, local concentration of binding molecules, or solvent, avoiding the need for harsh chemical treatments or the high temperatures required to remove chemisorbed molecules.^62,63^

**Figure 11:**
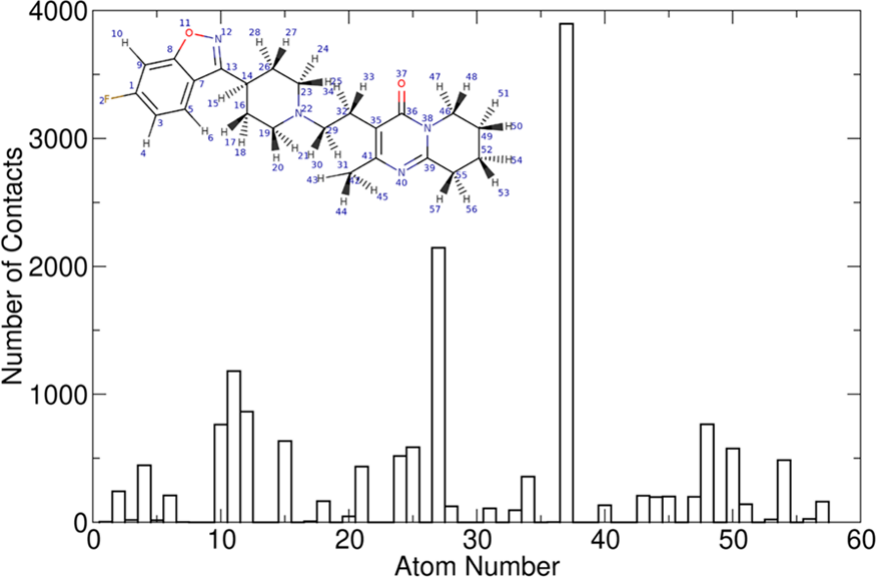
Computed number of closest contacts that each RIS atom makes with MCC during 32 ns of strong binding dynamics. The inset shows a sketch of RIS with the atoms numbered.

**Figure 12:**
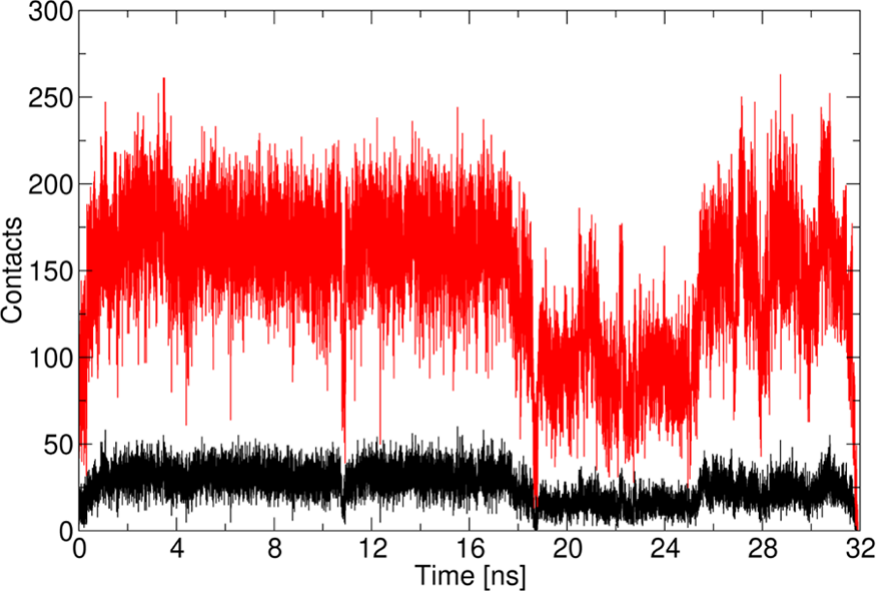
Total number of contacts formed between RIS and MCC during 32 ns of dynamics. The black line shows the number of contacts within 0.3 nm, while the red line shows the number of contacts within 0.4 nm.

Figure 11 shows how often a particular atom of RIS is closest to the MCC surface during the 32 ns of strong binding. The oxygen atom of the ketone group (atom 37) is the most frequent closest contact (see also the movie in the SI, which demonstrates the dynamic nature of the adsorption). The lone pairs of electrons make the *R*_2_C=O oxygen site an excellent H-bond acceptor. In addition, an in-plane hydrogen atom of the piperidine ring (atom 27) is ideally situated to interact with cellulose whenever atom 37 interacts with the surface. Atoms 11 and 12, the oxygen and nitrogen atoms of the 5-member ring also bind frequently to the MCC surface. All of these atoms are found on the same side of the molecule, and it is safe to assume that they constitute their binding face or “sticky” patch. Figure 12 shows the total number of contacts between RIS and MCC within a given distance, here 0.3 nm (black) and 0.4 nm (red).

Note that the contacts involve more than H-bonds. They correspond to pairs of atoms between the API and excipient in close proximity to each other and susceptible to interact *via* both electrostatic (polar atoms) and vdW intermolecular interactions. Figure 11 and the SI movie show how many sites are actually involved in the binding of such a molecule on MCC, beyond just HBA/HBD pairs. In molecular simulations, H-bonds emerge due to intermolecular electrostatic and vdW interactions when the hydrogen atom of a donor and the electronegative atom such as fluorine, oxygen, or nitrogen of an acceptor adopt a particular geometric configuration. For large-area contacts such as typical API–excipient complexes, other atoms beyond the H-bond pairs also interact by electrostatic, polarization, and vdW forces. H-bonds are the strongest intermolecular interactions (together with π–π stacking, where available between conjugated rings on the API and surface) and will therefore generally direct the orientation of the binding process. In particular, Figures 11 and 12 show that RIS binds during the 32 ns with two modes involving a different number of contacts. Due to the presence of many single bonds, the rings can rotate with respect to each other.

The most stable conformation involves (in addition to the strongly bound ketone group) HBA atoms 11 and 12 of the 5-membered ring, although the ring may occasionally rotate away from the surface. The less stable conformation (the minor population of ∼8 ns in duration, in the interval of 17–25 ns in Figure 12) sees the binding relying only on atoms 11 and 12, while the ring carrying the ketone group (atom 37) rotates away from the surface. These binding dynamics can be observed in the first 32 s of the movie of the MD trajectory provided in the SI (1 s playing time corresponds to 1 ns of dynamics). Hydrogen bonding *via* the ketone oxygen is also a regular feature of the adsorption configurations identified using the Monto Carlo scan (*e.g*., Figure 6A), which emphasizes its key role in stabilizing the API–excipient complex.

Finally, the binding free energy of RIS on MCC was estimated using the MM-PBSA (Poisson–Boltzmann) and MM-GBSA (generalized Born) methods. Both methods confirmed the favorable binding of RIS on MCC. The computed binding free energies of −50 and −39 kJ mol^–1^, respectively, indicate strong binding, though it is important to note that both approaches are known to overestimate free energies. This is because bulk dielectric constants approximate the full explicit solvent effect and the neglect or incomplete treatment of entropy. Taking these factors into consideration, the estimates are consistent with the 1–2 H-bonds of types C=O···HO and (O or N)···HO with binding energies of *ca*. −25 kJ mol^–1^ each plus some minor vdW contacts between the API and excipient.^64^ The numbers are also consistent with those obtained using the MC-based adsorption locator, given the differences in underlying model physics, parameter sets, and the consideration of binding to a large cellulose nanoparticle in methanol *vs* more flexible lactose disaccharide surface in vacuum.

## Discussion

This study was undertaken to test the findings of ref (34). The data presented in that work predicted that crystallization of a wide range of APIs (at low to modest supersaturation) is accelerated in the presence of a range of widely used excipients, all of which offer HBD sites. This acceleration is most pronounced when the technique is applied to APIs that do not themselves have HBD sites. The acceleration ranges from 4 to 16 times when the APIs without HBD capacities (listed in Table 1) are crystallized in the presence of MCC, compared to crystallization without an added heterosurface for the same supersaturation.^9,10,34^

The most significant finding from the simulations reported here is that the adsorption energies measured by the MC and MD methods are entirely consistent with literature values for small organic molecules generally adsorbed on metal oxides, in particular, silica surfaces.^36,38−41,65^

The treatment then of the first step in heterogeneous nucleation as an adsorption process offers useful insight into the mechanism, indicating that the binding free energies are very significant, predicted by MD to be approximately −50 kJ mol^–1^. A further point is that the adsorption energies for AAP adsorbed onto all of the surfaces of lactose examined here were all similar in magnitude (Table 2; MC scans). This indicates the primary importance of the chemical interaction, rather than the exact surface crystallography in the case of lactose with its abundance of HBD sites. This is also consistent with MD simulations, which show that the adsorption of RIS was not favorable on several of its surfaces, namely, (100) and (001), which lack the necessary and complementary hydrogen-bonding groups. On the complementary (010) surface of MCC, RIS makes strong, long-lived H-bonds and remains strongly bound for tens of nanoseconds.

The MC and MD simulations undertaken for this work are complementary, forming a neat workflow for rapid identification of possible binding sites followed by a detailed examination of selected major population(s). The adsorption locator module in the Materials Studio suite of programs uses a Monte Carlo approach to rapidly scan and estimate the strength of a broad range of adsorption modes. MC calculates thermodynamic statistical probabilities of acceptance/rejection of moves. By contrast, MD simulations generate a trajectory or time history of the system, which is followed over a period. In our case, we monitored interactions over 100 ns (strong binding persisted for ∼32 ns, followed by looser association for a further ∼65 ns; Figure 8). During the 32 ns of strong association, thousands of individual adsorption configurations are computed and these collectively can be used to estimate the adsorption time for RIS attached to an MCC surface in the presence of solvent molecules. The movie of the MD trajectory in the Supporting Information shows that the RIS molecule only completely detaches from the MCC surface during the final few nanoseconds of the 100 ns of dynamics. Instead of leaving after the ∼32 ns of strong binding, the molecule tumbles through a variety of weakly bound poses, which is consistent with the multiple adsorption configurations identified from the Monte Carlo work.

We also note that the MC computed adsorption enthalpies for APIs onto lactose *vs* onto their own crystal structures were similar, perhaps demonstrating why the heterosurface has been reported to behave almost as effectively as seed in accelerating crystallization.^34^

There are fewer literature reports where heterosurfaces without HBDs were examined.^6,15,31^ These were graphite and poly(caprolactone), neither of which generated a significant acceleration of the crystallization process. The MD simulations carried out here also indicate that HBD capacity in the heterosurface is a prerequisite for adsorption of the API molecules included in this study (which did not include APIs that exhibited only HBA capacities). In addition, MD simulations showed zero binding of RIS on the (100) and (001) surfaces of MCC, which all lack the HBD functionality.

**Scheme 1:**
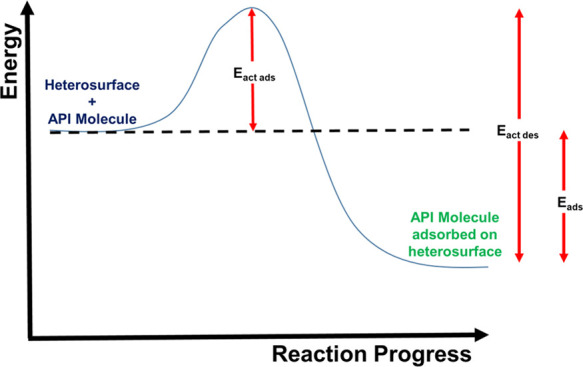
Energetics of Adsorption onto a Heterosurface

From an adsorption theory perspective, the adsorption lifetime comes about because the *E*_ads_ contributes to the size of the activation energy for desorption, as illustrated in Scheme 1. The adsorbed state represents a deep energy well. Escape (desorption) involves much higher activation energies than for the adsorption. The difference between the activation energy for adsorption (*E*_act ads_) and the activation energy for desorption (*E*_act des_) is the adsorption energy (*E*_ads_). The lifetime (τ) of adsorbed species is related to *E*_ads_ by eq 1(37,39,66−69)
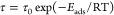
1

where τ_0_ is the lifetime of single molecular vibration. Literature values for τ_0_ are typically in the range of 10^–12^–10^–17^ s.^41,65^ Using this approach, we can estimate lifetimes of the adsorbed APIs (from the MC energies in Tables 2 and 3) of 10^–1^–10^14^ s. Clearly, these values are unreasonable. Equation 1 is usually applied to simple molecules (often diatomic) with a single clearly defined adsorption interaction. In the case of more complex molecules such as those examined here, there are multiple electrostatic and vdW adsorption interactions that collectively contribute to the overall *E*_ads_.

An alternative approach is to take the adsorption lifetime determined for RIS on MCC determined from the MD simulation. Applying this value (32 ns) to eq 1 yields an *E*_ads_ of −25 kJ mol^–1^. This value is in the range associated with 1–2 H-bonds and is approximately half the adsorption enthalpy of −55 kJ mol^–1^ calculated in MD simulations of limonene on SiO_2_.^39^ This approach would indicate that only the strongest binding interactions, namely, H-bonds, significantly contribute to the adsorption lifetime.

More generally, adsorption energies like lattice energies are typically made up of two components, namely, electrostatic and vdW interactions. There are numerous examples where the vdW forces constitute the major part of the lattice energy.^70,71^ The individual components of the overall vdW interactions are by definition tiny in size and therefore transitory in nature. By the same calculation presented above (eq 1), individual vdW interactions with energies less than 1 kJ mol^–1^ would return lifetimes on the order of a single molecular vibration (10^–12^–10^–13^ s). However, in the solid state, the time constant for translation would be many orders of magnitude greater than the time constant for the switching on and off of transient vdW forces. Therefore, in the context of the solid state where the interacting compounds are not translationally free, vdW forces become an important part of the total lattice energy. Here, we argue that the vdW component of adsorption energies switches on and off too quickly to affect translation or diffusion away from a heterosurface and does not contribute to the lifetime of the adsorbed species. This then leaves just the role of hydrogen bonding to be explored, consistent with the growing experimental reports of heterocrystallization.^11−34^

Central to our hypothesis on the action of heterosurfaces is the concept that adsorbed molecules have longer lifetimes (on the order of tens of nanoseconds) than molecule-to-molecule interactions in solution (on the order of picoseconds). Here, we present clear evidence that we can expect API molecules adsorbed onto suitable heterosurfaces to exhibit adsorption lifetimes in excess of 10 ns. In an earlier publication, we estimated the time required to add a single molecule of an API to a growing crystal to be on the order of 1–50 ps.^34^ The simulation data we report here confirms that a typical API adsorbed onto a typical heterosurface has a lifetime that is several orders of magnitude longer than the time required to add a single or multiple molecules or clusters of molecules of the API to a growing nucleus or cluster.

Our postulated mechanism of heteronucleation involves the adsorption of APIs onto sites on the heterosurface at which they remain attached for an extended time scale (tens of nanoseconds). By contrast, the time scale of API–API collisions in homogeneous solution leading to homogeneous nucleation is of the order of tens of picoseconds, limiting the time available for attachment of further API molecules. When attached by adsorption to a heterosurface, the API molecule can experience multiple collisions with other API molecules from solution, some of which will also be aided by a favorable enthalpy of adsorption and grow by multipole additions toward the formation of a critical nucleus size. The growth to the critical nucleus size need not necessarily be *via* the addition of single API molecules but may also involve growth to the critical size by cluster addition to the adsorbed API.^72^

The total adsorption energy then provides only a first approximation of the lifetime of the adsorbed species. Of more importance is the presence of strong hydrogen bonding between the heterosurface and the API. In the absence of complementarity between HBD and HBA capacities on the API and the heterosurface, strong hydrogen bonds are not formed and the API rapidly dissociates from the heterosurface. Strong hydrogen bonding anchors the API onto the heterosurface while allowing vibrational motions and exchanges between equivalent hydrogen-bonding sites (without unbinding) that may facilitate favorable positioning of additive molecules or clusters from the crystallizing solution as the ordered, well-formed nucleus assembles.

## Conclusions

Atomistic computer simulations of API molecules adsorbed on excipient surfaces identified strong modes of adsorption and adsorption energies entirely consistent with literature studies of the adsorption of small organic molecules on metal oxide surfaces. The lifetime of APIs adsorbed onto excipients in methanol solution has been determined most precisely for RIS adsorbed onto MCC using MD simulations. The MD-calculated hydrogen-bonded lifetime of 32 ns is long in comparison with the lifetime of API–API collision and attachment in solution. It is orders of magnitude longer than the time required for the addition of a single molecule to a growing crystal at low to moderate levels of supersaturation. A mechanism of heteronucleation is presented, whereby API molecules attached to a heterosurface can experience multiple collisions during tens of nanoseconds with API molecules or clusters from homogeneous solution, sufficient to facilitate growth to a critical nucleus size. This mechanism postulates that the extended lifetime of adsorbed API is an essential feature of efficient, reproducible heteronucleation.

## Supporting Information Available

Consists of a movie showing the MD trajectory of the risperidone (RIS) molecule binding to the microcrystalline cellulose (MCC) surface. The movie reveals that the RIS molecule only completely detaches from the MCC surface during the final few nanoseconds of the 100 ns of dynamics. Instead of leaving after the ∼32 ns of strong binding, the molecule tumbles through a variety of weakly bound poses, which is consistent with the multiple adsorption configurations identified from the Monte Carlo work. This movie is available online (MP4)
